# Engineering of Silane–Pyrrolidone Nano/Microparticles and Anti-Fogging Thin Coatings

**DOI:** 10.3390/polym16142013

**Published:** 2024-07-14

**Authors:** Natalie Mounayer, Shlomo Margel

**Affiliations:** Department of Chemistry, Bar-Ilan Institute of Nanotechnology and Advanced Materials, Bar-Ilan University, Ramat-Gan 5290002, Israel; natali_monayer@hotmail.com

**Keywords:** silane–pyrrolidone monomer, nanoparticles, microparticles, Stöber polymerization, thin films, anti-fogging coatings

## Abstract

Polyvinylpyrrolidone (PVP) exhibits remarkable qualities; owing to the strong affinity for water of its pyrrolidone group, which enhances compatibility with aqueous systems, it is effective for stabilizing, binding, or carrying food, drugs, and cosmetics. However, coating the surface of polymeric films with PVP is not practical, as the coatings dissolve easily in water and ethanol. Poly(silane–pyrrolidone) nano/microparticles were prepared by combining addition polymerization of methacryloxypropyltriethoxysilane and N-vinylpyrrolidone, followed by step-growth Stöber polymerization of the formed silane–pyrrolidone monomer. The silane–pyrrolidone monomeric solution was spread on oxidized polyethylene films with a Mayer rod and polymerized to form siloxane (Si-O-Si) self-cross-linked durable anti-fog thin coatings with pyrrolidone groups exposed on the outer surface. The coatings exhibited similar wetting properties to PVP with significantly greater stability. The particles and coatings were characterized by microscopy, contact angle measurements, and spectroscopy, and tested using hot fog. Excellent anti-fogging activity was found.

## 1. Introduction

In the modern age, there has been a garnered increasing interest in the advancement of surface modification techniques aimed at imparting specific functional properties to a variety of materials [1,2,3,4,5,6]. The modified properties of the materials have enabled the development of materials with tailored properties, facilitating their use in various industrial and agricultural applications [7,8,9,10]. The development of durable thin coatings on material surfaces, in particular, polymeric surfaces, has gained significance in recent years. Such development is particularly important for addressing challenges related to fogging. Fogging is prevalent in various sectors, including the automotive industry, medical devices, agriculture, and various packaging industries [4,11,12,13,14,15,16].

Fogging is caused by the condensation of water vapor into droplets on surfaces, resulting in light scattering, which can severely impact the visibility of clear materials and impede their functionality [15]. The development of anti-fog coatings on the surface of materials has been an issue undergoing extensive research, including exploring various approaches to enhance the durability and effectiveness of these coatings. In this context, polymers demonstrate diverse properties influenced by their functional groups, impacting the morphology and surface of the coated material. Polymeric coatings may thus be fine-tuned, facilitating the selection of inherent material characteristics with the stringent requirements of specific applications [17]. Early attempts involving the use of hydrophilic polymers such as polyvinylpyrrolidone (PVP) suffered from poor adhesion and durability despite the effectiveness in reducing fogging [14].

PVP derived from N-vinyl pyrrolidone (VP) stands out among polymers, having exceptional qualities. The hydrophilic nature of PVP, derived from its pyrrolidone groups, enables a wide range of applications, including pharmaceuticals, cosmetics, and agrochemicals [18,19,20,21]. It improves dispersion, dissolution, and adhesion and enhances the bioavailability of active ingredients [22,23,24]. Despite these benefits, PVP attached to material surfaces through physical bonds lacks durability, requiring alternative approaches to achieve durable coatings [25].

This limitation was recently addressed by researchers using organosilane materials, which have the ability to form robust covalent bonds with the surface substrate, leading to significantly enhanced coating stability and durability [26]. This study aims to simplify the earlier sophisticated approach [27] and expand its functionality. This is attributed primarily to the effect on the surface and morphology of functional groups, leading to self-cross-linking and covalent bonding [26].

This novel approach to anti-fog coatings through step-growth polymerization on oxidized surfaces of polymeric films focusing on polyethylene (PE) is simple, cost-effective, and environment-friendly [28]. Silane-based coatings, such as those derived from methacryloxypropyltriethoxysilane (MPTES), have shown promise due to their ability to form siloxane (Si-O-Si) self-cross-linked networks [29]. The siloxane cross-linking and covalent bonds between the coating and oxidized polymer surface not only improve the mechanical stability and durability of the coatings, but also retain the hydrophilic properties necessary for the formation of anti-fog coatings [15,30]. Moreover, both SiO_2_ and PVP are prevalent, inexpensive, non-toxic, and food-safe [31].

**Figure 1:**
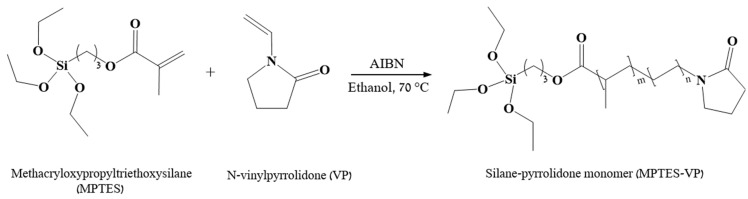
Synthesis of silane–pyrrolidone monomer from MPTES and VP.

**Figure 2:**
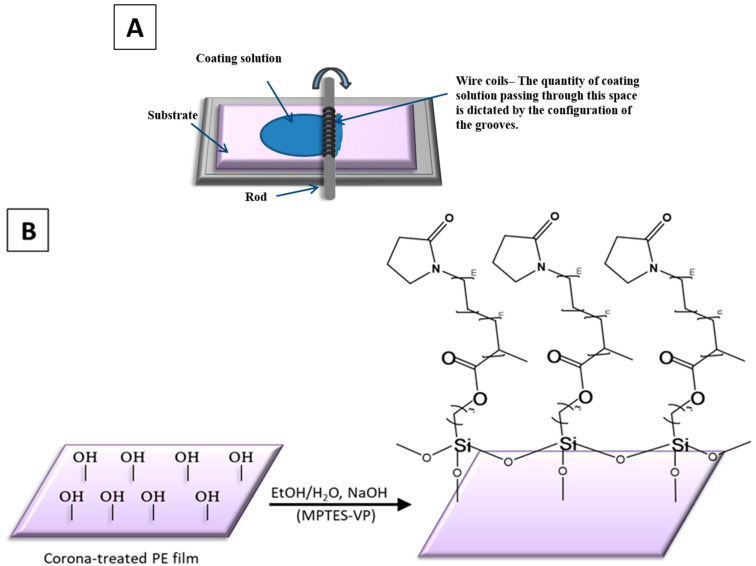
Mayer rod coating system (**A**) and P(MPTES-VP) thin coating of PE film (**B**).

The combination of VP with MPTES through polymerization has been a particularly effective strategy, resulting in the formation of the combined monomer MPTES-VP (Figure 1). P(MPTES-VP) nano/microparticles were subsequently synthesized through aging step-growth polymerization [32]. Thin coatings were obtained by spreading the monomeric dispersion onto polyethylene films which underwent oxidation with a Mayer rod and were polymerized, followed by polymerization on the surface of the substrate in basic conditions [33] (Figure 2B). Extensive characterization and testing procedures were performed on both the particles and the coatings which analyzed their physical, chemical, and morphological attributes. The coatings underwent testing procedures including the hot-fog test and a durability evaluation.

## 2. Experimental Section

### 2.1. Materials

Analytical grade chemicals procured from Sigma-Aldrich (Rehovot, Israel) included ethanol (EtOH, anhydrous, HPLC grade), VP, and sodium hydroxide (NaOH). J&K Scientific Ltd. (Guangzhou, China) supplied MPTES. Azobisisobutyronitrile (AIBN, Sigma-Aldrich, Rehovot, Israel) underwent recrystallization using methanol to achieve the desired purity. The double-distilled water (DDW) used in the experiments was produced by a TREION purification system (Tel-Aviv, Israel). Poleg Ltd. (Kibutz Gevim, Israel) provided the surface-oxidized PE films.

### 2.2. Methods

#### 2.2.1. Surface Oxidation of Polyethylene Films

Treatment by corona (Vetaphone Corona & Plasma, Kolding, Denmark, 250 W·min/m^2^) oxidized the polymeric film surface to improve adhesion and enhance surface energy [15]. This treatment promotes the improved adhesion of subsequent coatings by introducing polar functional groups onto the surface.

#### 2.2.2. Preparation of PVP

PVP was prepared through radical polymerization of VP with a molecular weight of 111.14 g/mole. Polymerization was carried out for 24 h at 70 °C (18 *v*/*v*%) under an N_2_ atmosphere using AIBN (0.2 *w*/*v*%) as the initiator in the ethanol (1 mL) continuous phase.

#### 2.2.3. Preparation of Silane–Pyrrolidone Monomer

MPTES (3 *v*/*v*%) and VP (15 *v*/*v*%) underwent radical co-polymerization at 70 °C in an N_2_ atmosphere for 24 h in the presence of AIBN (0.2 *w*/*v*%) in the ethanol (1 mL) continuous phase. The resultant monomer is termed MPTES-VP (Figure 1).

#### 2.2.4. Synthesis of Poly(silane–pyrrolidone) Nano/Microparticles

Nanoparticles and microparticles with a range of diameters were afforded by step-growth polymerization (Stöber process) of the formed MPTES-VP monomer. Polymerization involved mixing 100 μL of a 1 mM NaOH aqueous solution with 500 μL of the MPTES-VP ethanol solution. The mixed solution was left to age for different periods of time (1, 5, 30, and 60 min) and the diameters and diameter distributions of the formed P(MPTES-VP) particles were determined by TEM and HRSEM.

#### 2.2.5. Poly(silane–pyrrolidone) and PVP Thin Coatings on PE Films

Oxidized polyethylene (PE) films were thinly coated with P(MPTES-VP) (Figure 2B) by polymerizing the synthesized monomeric solution (MPTES-VP) and then spreading it with a Mayer rod [34] (Figure 2A) to achieve a 6 μm thickness (RK Print Coat Instruments Ltd., Litlington, UK). Briefly, the synthesized monomeric solution (MPTES-VP EtOH (500 μL)) was mixed with 1 mM NaOH (100 μL)) to induce polymerization, followed by spreading with a Mayer rod and air-drying. The coatings were thenwashed with ethanol to remove excess reagents and air-dried at 80 °C for 5 min. PVP thin coatings were applied in a similar manner, by spreading the PVP EtOH (500 μL)-H_2_O (100 μL) solution on oxidized PE films, air-drying, washing with ethanol, and air-drying at 80 °C for 5 min.

### 2.3. Characterization of Silane–Pyrrolidone Nano/Microparticles

#### 2.3.1. High-Resolution Scanning Electron Microscopy (HRSEM) and Energy-Dispersive X-ray Spectroscopy (EDS)

Free P(MPTES-VP) nano/microparticles dispersed in EtOH were purified from excess reagents by centrifuging at high speed (14,000 rpm, 10 min) and redispersed in EtOH three times; a drop of the dispersion was spread evenly on a silicon wafer and dried at room temperature for 48 h. The wafer was coated with iridium in a vacuum prior to viewing by HRSEM (Magellan FEI 400 L, 5 kV, Hillsboro, USA) in addition to an energy-dispersive X-ray spectroscopy detector (Oxford).

#### 2.3.2. Transmission Electron Microscopy (TEM)

The nanoparticles and microparticles were structurally characterized (size and morphology) with a JEM-1400 instrument (JEOL, 120 kV, Tokyo, Japan). As described above, P(MPTES-VP) particles in EtOH/water were centrifuged (14,000 rpm, 10 min) and redispersed in ethanol repeatedly; a drop was placed on a copper grid and air-dried for 48 h.

#### 2.3.3. X-ray Photoelectron Spectroscopy (XPS)

Surface elemental analysis of the films was performed using a Nexsa spectrometer (Thermo Fisher Scientific, Manchester, UK), featuring a monochromatic micro-focused low-power Al Kα X-ray source with a photon energy of 1486.6 eV. Survey and high-resolution spectra were obtained at pass energies of 200 and 50 eV, respectively, with the source operating typically at 72 W. The binding energies were recalibrated by setting the C 1s peak CC/CH component to 285 eV unless specified otherwise.

Quantitative chemical analysis of the surface was conducted using high-resolution core-level spectra after removing a nonlinear smart background. The measurements were executed under ultra-high vacuum conditions with a base pressure of 5 × 10^−10^ torr, not exceeding 3 × 10^−9^ torr. Vision 2 Software (Kratos, Manchester, UK) was employed for spectral analysis and deconvolution, with overlapping signals resolved into Gaussian/Lorentzian components.

### 2.4. Characterization of Poly(Silane–Pyrrolidone) Anti-Fog Thin Coating

#### 2.4.1. Hot-Fog Test

The assessment of anti-fogging properties was conducted using a hot-fog test that simulates real conditions of fog formation [33]. Briefly, a vial (20 mL) containing 5 mL of water was heated to 70 °C. The film was then secured to the opening of the vial, with the coated side facing the water. Regularly observation of water condensation on the coated film provided the necessary visibility for evaluating the anti-fogging activity of the coatings.

#### 2.4.2. Ultraviolet–Visible (UV-Vis) Spectroscopy

UV–Vis spectra (200–600 nm) of the coated and uncoated films were obtained using a spectrophotometer (Cary 5000, Agilent Technologies, transmission mode, Santa Clara, CA, USA).

#### 2.4.3. Contact Angles (CAs)

Static water contact angles were measured with a goniometer (OCA20, Data Physics Instruments, Filderstadt, Germany). DDW sessile drops (5 μL) were deposited on four designated areas of the coated and uncoated films, and images of the drops on the film were taken after a few seconds and fitted to Laplace–Young curves.

#### 2.4.4. Fourier-Transform Infrared (FTIR) Spectroscopy

FTIR spectra were obtained using an Alpha-FTIR (Bruker, Billerica, MA, USA) Quick Snap™ module for sampling with a platinum attenuated total reflectance (ATR) diamond module.

#### 2.4.5. Atomic Force Microscopy (AFM)

AFM imaging was conducted with a Bio FastScan scanning probe microscope (Bruker AXS, Billerica, MA, USA) in PeakForce QNM mode with a Bruker FastScan-C probe (silicon, 0.45 N/m spring) in an acoustic hood. Images were obtained in the retrace direction (1.7 Hz, 512 samples per line), processed, and analyzed for roughness (Nanoscope Analysis 1.5) employing the “planefit” and “flatting” functions.

#### 2.4.6. Coating Durability

The durability of the films was assessed by conducting a tape test, after washing them with ethanol followed by water, wherein an adhesive tape was pressed firmly onto the film and slowly peeled off, as described [35]. The procedure was repeated (10 and 20 times) and the wettability of the coating was ensured by checking the water CA. The films were dipped in EtOH (3 times) and DDW and dried in air. The dry films were analyzed by AFM and HRSEM and the anti-fog activity was evaluated with hot fog.

## 3. Results and Discussion

### 3.1. P(MPTES-VP) Nano/Microparticles

First, the MPTES-VP monomer, shown in Figure 1, was prepared by means of addition polymerization of MPTES and VP with an AIBN initiator in an EtOH continuous phase, as described in Section 2.2.3. In the next stage, nano- and microparticles with different sizes and size distributions were prepared by Stöber polymerization. In this process, self-cross-linking of the MPTES-VP monomer occurred in an EtOH-H_2_O solution. The extent of the cross-linking varied according to the aging duration (Section 2.2.4; the extent increased with aging).

### 3.2. Microscopy (HRSEM, TEM, EDS, and XPS)

**Figure 3:**
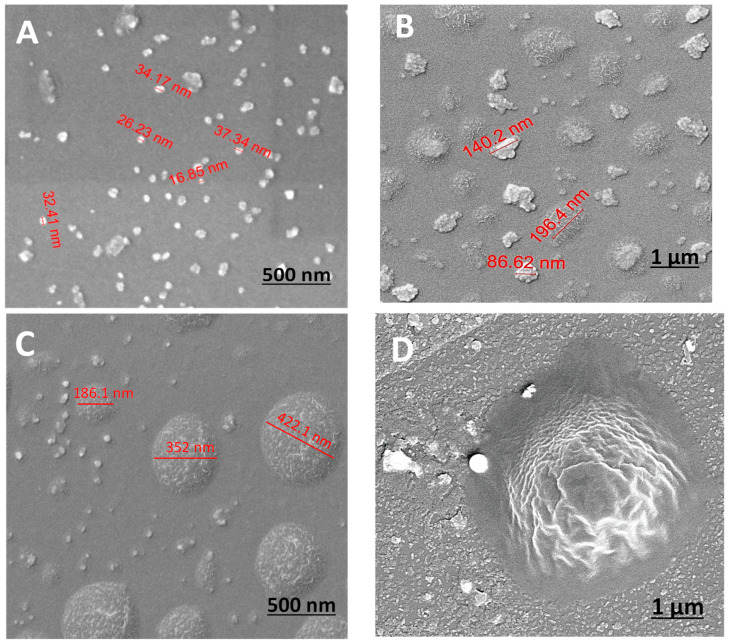
HRSEM images of P(MPTES-VP) particles after 1 (**A**), 5 (**B**), 30 (**C**), and 60 min (**D**).

HRSEM surface images (Figure 3) were used to evaluate the diameters of the particles as well as the surface morphology. HRSEM revealed variations in topography, illustrating the evolution of particle structure over time. Figure 3 shows that the obtained particles do not exhibit a distinct round shape, and over time, the range of diameters increases from nano to micron size. Initially, relatively monodispersed nano-sized particles were observed in the first minute, ranging from 15 to 50 nm in diameter (Figure 3A,B). However, after 5 and 30 min, micron-sized particles of a very broad size distribution (between 0.2 and 0.7 μm) were also observed (Figure 3B,C), accompanied by the noticeable presence of aggregates. Figure 3D illustrates the clear morphology of the PVP coating layer on a micron-sized particle after 1 h.

It is crucial to note the diverse sizes and structure distributions at every time point, with increasing diversity over time. The changes in particle diameter are due to the coalescence in the growth process of the siloxane polymerization [32]. As the process advances, particles tend to expand and merge, leading to a broader range of sizes. Initially, nucleation results in the formation of small, uniform nanoparticles. As the reaction continues, these particles aggregate and grow, leading to the formation of larger, less uniform particles. Polymerization makes the surface of the particles more complex, further contributing to the observed diversity in size and morphology.

This observation is corroborated by research indicating that the growth of silica particles during polymerization is time-dependent [36]. The hydrolysis and condensation of tetraethyl orthosilicate (TEOS) in the presence of a catalyst results in the formation of silica particles that increase in size over time due to further condensation reactions. Other studies have demonstrated that the particle size distribution becomes broader with extended reaction times [37], aligning with the observed HRSEM results.

**Figure 4:**
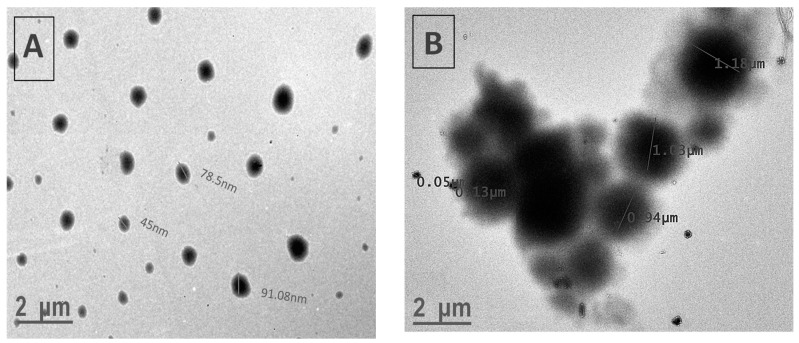
TEM images of P(MPTES-VP) particles obtained after 1 min (**A**) and 1 h (**B**).

**Figure 5:**
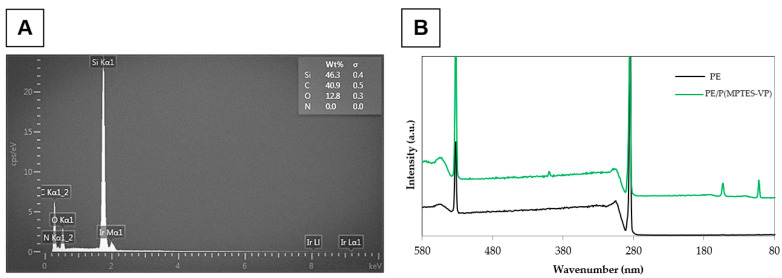
EDS (**A**) and XPS (**B**) of PE alone and coated with P(MPTES-VP) particles (60 min of aging).

Figure 4 exhibits TEM images of P(MPTES-VP) particles after 1 min and 1 h. The particle diameter clearly increases during polymerization. This can be attributed to the significant effect of coalescence in the growth process, which influences the observed increase in particle size distribution, as discussed earlier. This core–shell (inorganic/organic) structure is absent in the nano-sized particles (Figure 4A). It is important to highlight that TEM images of micron-sized particles clearly show a core of inorganic material (SiO_2_) covered with an organic layer (Figure 4B), supported by the results of the EDS and XPS analysis. The EDS analysis (Figure 5A) indicates the presence of the chemical elements Si, C, and O, which comprise the core of the particles, along with the presence of an iridium (Ir) peak, attributed to the spraying of the conductive layer onto the fibrous mats to improve conductivity during HRSEM examination. Elemental nitrogen did not appear in the EDS analysis, as it appears in the pyrrolidone ring, which is present in the outer shell.

This EDS procedure is suitable for thin-shell surface characterization, as it penetrates only to the inner content of the particles (core) and is not sensitive enough to reach the outer shell, which appears in the XPS analysis. The analysis presented in Table 1 and Figure 5B exhibits the prominent peaks of the surface-oxidized PE at 287 eV for C 1s and 535 eV for O 1s, with an atomic concentration (wt%) of 88.45 and 15.23%, respectively. Surprisingly, a trace amount of N 1s was observed at 405 eV (0.07 wt%), likely contributed from environmental contamination.

Moreover, the XPS analysis of the P(MPTES-VP) coating exhibits an additional peak at 101 eV for Si 2p (2.77 wt%) and an increased atomic concentration for O 1s and N 1s (23.18 and 5.31 wt%), indicating the presence of the P(MPTES-VP) coating on the surface of the oxidized PE film. These results are aligned with the reported literature in which organosilane compounds are known to form a silica core with an organic polymer outer shell [38]. Organosilanes undergo hydrolysis and condensation reactions to form a silica framework. This framework acts as a durable core, while the organic functional groups on the silane molecules participate in further polymerization, resulting in the formation of an outer organic polymer layer.

### 3.3. P(MPTES-VP) Anti-Fogging Durable Thin Coatings on PE Films

The preparation of P(MPTES-VP) thin coatings on corona-treated PE films was carried out by spreading the monomeric silane–pyrrolidone (MPTES-VP) solution on the oxidized surface of the films. This was performed using a Mayer rod coating system [34] (Figure 2A) with a wet deposit thickness of 6 μm. This was followed by polymerization as described in Section 2.2.5 (Figure 2B). The coatings underwent microscopy including AFM, TEM, and HRSEM. Varius spectroscopic methods were employed including UV-vis and FTIR/ATR spectroscopy and XPS. In addition, sessile water drop contact angle measurements were carried out. Hot-fog and durability tests were performed. The durability of the coating on the surface of the oxidized film is probably attributed to the cross-linking siloxane bonds formed between the MPTES-VP monomers and the hydroxyl surface groups. This cross-linking is formed by the covalent bonding between the monomers and the oxidized film surface.

### 3.4. Hot-Fog Test

**Figure 6:**
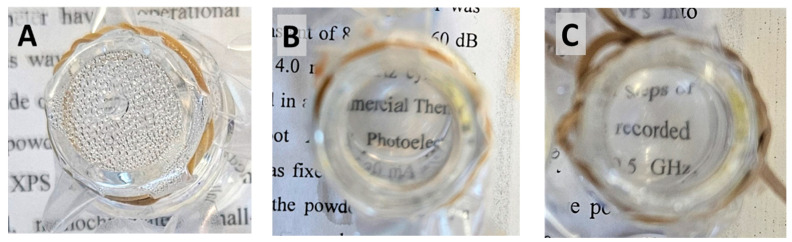
Hot-fog test: digital images of non-coated (**A**), P(MPTES-VP)-coated (**B**), and PVP-coated (**C**) PE films 1 h after water in vial reached 70 °C.

Figure 6 exhibits the hot-fog test results of non-coated PE films (used as a control) and films coated with a distinct thin layer of P(MPTES-VP) and PVP. These experiments were employed to replicate conditions that are conducive to fog formation on the PE surface, as outlined in Section 2.4.1. Images displaying the anti-fogging characteristics of the films were captured one hour subsequent to the water in the vial reaching 70 °C. As shown in Figure 6, the PE film (Figure 6A) exhibits a foggy surface throughout the experiment. However, the anti-fog film, coated with P(MPTES-VP), demonstrates outstanding transparency (Figure 6B), maintaining this clarity in all measurements conducted over 24 h. On the contrary, the transparency of the PVP coating (Figure 6C) gradually decreases over time, and after 24 h, it becomes comparable to that of the non-coated film due to the dissolution in water over time. These results indicate an elevated surface energy and enhanced hydrophilicity of the surface of the coated films, enabling the dispersion of water droplets due to the properties of pyrrolidone.

### 3.5. Ultraviolet–Visible Spectroscopy (UV-Vis)

**Figure 7:**
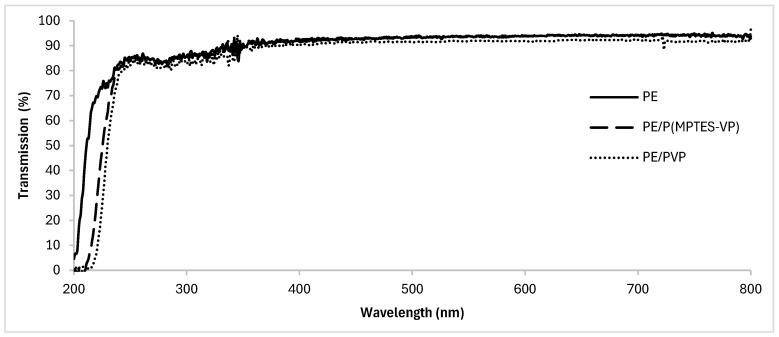
UV–visible transmission spectra of oxidized PE, PE/PVP, and PE/P(MPTES-VP) films.

UV-Vis spectroscopy of the coated and uncoated films (Figure 7) shows that the coatings slightly shifted the transmission of UV light; importantly, they maintained the transmission of UV-visible light through the film and did not significantly affect the optical transparency. High optical transparency is essential for plastic films, rendering them suitable for applications requiring both anti-fog properties and optical clarity. These properties are desired in the food, medical, and plastic packaging industries.

### 3.6. Contact Angles (CAs)

Surface wettability was assessed by measuring water sessile CAs to analyze the hydrophilicity of the coating. These CAs are affected by both the chemistry and the surface roughness. In general, rougher surfaces and the presence of hydrophobic groups contribute to higher CAs, whereas smoother surfaces and hydrophilic groups tend to produce lower angles. The oxidized PE film had a CA of 69 ± 3°, while PE/P(MPTES-VP) exhibited a significantly lower CA of 16 ± 0.7°, similar to that of freshly prepared PE/PVP (18 ± 1°). These results reflect the hydrophilic nature of the coatings owing to the presence of pyrrolidone groups in comparison with the more hydrophobic surface of the oxidized film.

### 3.7. Fourier-Transform Infrared Spectroscopy with Attenuated Total Reflection (FTIR/ATR)

**Figure 8:**
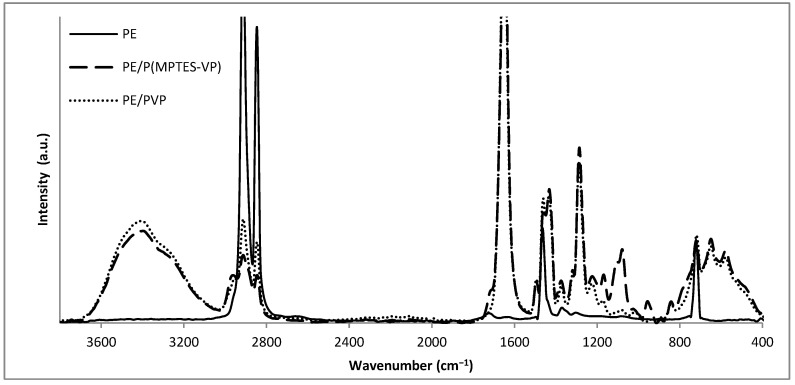
FTIR/ATR spectra of oxidized PE, PE/P(MPTES-VP) and PE/PVP films.

The elemental composition of the coated films was evaluated using FTIR/ATR (Figure 8) to verify the presence of the specific coating components and show the modifications in surface composition. The distinctly visible peaks at 2850, 2917, 1473, and 731 cm^−1^ (attributed to C-H asymmetric and symmetric stretching vibrations of methylene groups, C-H bending, and C-C rocking, respectively) correspond to the characteristic absorption peaks of the PE film in all spectra. Figure 8 reveals the Si-O-Si peaks at 1074 and 1091 cm^−1^ (indicative of asymmetric stretching vibration) and a moderated band at 955 cm^−1^ (associated with the bending of the linear Si-OH stretching vibration) [15,39] for the PE/P(MPTES-VP) coating. The peak at 1724 cm^−1^ corresponds to the carbonyl group in the acrylate component of MPTES. In addition, the PVP control exhibits prominent peaks at 1286 and 1650 cm^−1^, which correspond to the C-N stretching and carbonyl amide group of the pyrrolidone ring [40], respectively.

### 3.8. Atomic Force Microscopy (AFM)

The comparative surface roughness (Rq) of the corona-treated PE and coated PE films was assessed by AFM analysis. The Rq of the corona-treated and PVP-coated films was 26 ± 3 and 15 ± 4 nm, respectively, whereas the P(MPTES-VP) coating significantly reduced the roughness to 6 ± 3 nm. These findings align well with the low contact angle of the coated film in comparison with the pristine film. Additionally, the AFM results are well correlated with the observed anti-fogging activity.

### 3.9. Durability of P(MPTES-VP) Coating

Durability tests on the MPTES-VP-coated PE film involved tape tests and ethanol–water washing. The tape test showed stable anti-fogging properties with CAs of 16 ± 1, 16 ± 2, and 20 ± 6° (0, 10, and 20 times, respectively). Furthermore, PE/P(MPTES-VP), subjected to ethanol and water washing, exhibited consistent anti-fogging efficacy in the hot-fog tests. Analyses by FTIR/ATR, AFM, and HRSEM of the washed film yielded results identical to those observed before washing.

The silane–pyrrolidone monomer solution remained stable for a number of months. Upon polymerization and application onto corona-treated PE, following the procedure outlined in Section 2.2.4, the coating maintained comparable properties to those of the freshly prepared monomer. This highlights the material durability of the prepared coating, emphasizing self-siloxane cross-linking and covalent bond formation between the coating and the corona-treated PE surface groups.

It should be noted that the freshly prepared PVP coating on the oxidized PE film, prepared as described in Section 2.2.5, presented anti-fogging properties in the hot-fog test. However, within a short period of time, the PVP coating dissolved gradually. This PE/PVP film was also not sufficiently stable for washing with water or EtOH.

## 4. Summary and Conclusions

Silane–pyrrolidone monomers were prepared through addition copolymerization of MPTES and VP in ethanol. Core–shell (SiO_2_/PVP)-structured nanoparticles and microparticles with a range of diameters were afforded by using the Stöber step-growth polymerization process of the formed MPTES-VP monomer with aging for different periods of time. On the other hand, coatings were achieved by spreading the MPTES-VP monomer solution shortly after mixing with the basic solution on oxidized PE films with a Mayer rod system during surface polymerization between MPTES-VP and the substrate.

The P(MPTES-VP) coating demonstrated excellent durable anti-fogging properties validated by several tests. The hot-fog test exhibited the anti-fogging effectiveness, while UV-visible spectroscopy demonstrated that the coatings did not impact the PE film’s transparency. The coatings exhibited a low water contact angle (16 ± 0.7°) and low surface roughness (6.4 ± 2.7 nm), which contribute to their anti-fogging capabilities. Furthermore, PVP exhibits notable qualities, in particular, excellent adhesion properties and excellent hydrophilicity, enhancing the solubility and compatibility of PVP organosilane-based materials in aqueous systems. This approach offers a novel alternative to non-covalent binding of PVP onto polymeric surfaces, which is typically less stable and more prone to dissolution in aqueous conditions.

We intend to expand this work to include different polymeric films, such as polyvinylchloride (PVC), polypropylene (PP), polymethylmethacrylate (PMMA(, and polycarbonate (PC). Agricultural application and haze and clarity examinations of the P(MPTES-VP) nano/microparticles shall be explored. For example, the encapsulation of fungicides in the particles may allow for more effective pest control than the spraying of the aqueous dispersion. Encapsulation could offer a controlled release mechanism, providing sustained protection against pests and reducing the need for frequent reapplication. Such advancements and approaches could lead to more cost-efficient, environment-friendly, and simpler preparation procedures for a wide range of applications.

## Figures and Tables

**Table 1:** Quantitative surface XPS analysis of non-coated and coated PE films.

Film	Atomic Concentration (wt%)			
	Si 2 p	C 1 s	O 1 s	N 1 s
PE	-	88.45	11.48	0.07
PE/P(MPTES-VP)	2.76	71.07	20. 85	5.3

## Data Availability

The data are contained within this article.
